# The effects of seed detectability and seed traits on hoarding preference of two rodent species

**DOI:** 10.1111/1749-4877.12626

**Published:** 2022-01-24

**Authors:** Minghui WANG, Xianfeng YI

**Affiliations:** ^1^ College of Life Sciences Qufu Normal University Qufu China

**Keywords:** *Apodemus chevrieri*, *Leopoldamys edwardsi*, seed detectability, seed hoarding preference, seed traits

## Abstract

Seed traits play an important role in affecting seed preference and hoarding behaviors of small rodents. Despite greatly affected by seed traits, seed detectability of competitors represents pilfering risks and may also modify seed hoarding preference of animals. However, whether seed traits and seed detectability show consistent effects on seed hoarding preference of animals remain largely unknown. Here, we explored how seed traits and seed detectability correlate with seed hoarding preference of *Leopoldamys edwardsi* and *Apodemus chevrieri* in a subtropical forest. Despite the effects of seed coat thickness and caloric value on hoarding preference of *L. edwardsi*, we detected no significant effects of other seed traits on hording preference of the 2 rodent species. There was no correlation between larder‐hoarding preference and inter‐ or intra‐specific seed detectability of *L. edwardsi*; however, seed detectability of *L. edwardsi* was negatively correlated with its own scatter‐hoarding preference. Although scatter‐hoarding preference of *A. chevrieri* was not correlated with inter‐ and intra‐specific seed detectability, larder‐hoarding preference of *A. chevrieri* was positively correlated with intra‐specific seed detectability. Our study may provide evidence that intra‐specific seed detectability rather than seed traits and inter‐specific pilfering risks play an important role in modifying seed hoarding preference of rodents.

## Graphical abstract

Although seed detectability of competitors is greatly affected by seed traits, whether seed trait and seed detectability show consistent effects on seed hoarding preference of animals remain largely unknown. Here, we explored how seed traits and seed detectability correlate with seed hoarding preference of *Apodemus chevrieri* and *Leopoldamys edwardsi* in a subtropical forest. Our study may provide evidence that intra‐specific seed detectability rather than seed traits and inter‐specific pilfering risks play an important role in modifying seed hoarding preference of rodents.

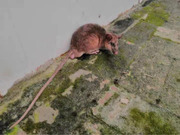

## INTRODUCTION

Food hoarding is a universal behavior of many rodents during periods of food shortage caused by changes in the environments (Dittel & Vander Wall 2018). Hoarding behavior is most frequent during autumn when a large number of seeds ripen (Vander Wall *et al*. 2018). In general, rodents exhibit 2 primary hoarding strategies: scatter‐hoarding and larder‐hoarding. Scatter‐hoarding animals store seeds in many small caches throughout their home range; while larder‐hoarding animals deposit food items they collected into a single location, such as a rock crack or a burrow chamber within a limit area (Vander Wall 2010; Yi *et al*. 2019). Scatter‐hoarding by animals plays a crucial role in seed dispersal and plant regeneration and provides mutual benefits to various tree species in many forest ecosystems (Steele *et al*. 2015; Vander Wall 2019).

In nature, trees usually produce seeds differing in a number of seed traits, such as seed size, hardness of seed coat, nutrient content, which in turn affect seed hoarding preference by scatter‐hoarding animals. Variations in seed traits have been verified to cause differences in hoarding preference of animals (Wang *et al*. 2014; Lichti *et al*. 2017). Seed fates can also be affected by seed traits through manipulating hoarding preference of rodents (Sundaram *et al*. 2018; Vander Wall *et al*. 2018). An increasing literature has accumulated that large seeds or seeds with a hard coat are more likely cached by animals because they require more time to handle them (Zhang & Zhang 2008; Chang *et al*. 2012; Jorge *et al*. 2012). High tannin hypothesis states that hoarding animals prefer to eat low‐tannin seeds but hoard high‐tannin seeds for later consumption because tannins hinder food digestion by animals (Xiao *et al*. 2008). Therefore, previous studies have provided solid evidence that seed trait is a faithful indicator in determining seed hoarding preference of rodents (Steele *et al*. 2006; Wang & Chen 2009; Xiao *et al*. 2013; Sundaram *et al*. 2018).

However, food items scatter‐hoarded by animals are often pilfered by intra‐ or inter‐specific competitors (Jansen *et al*. 2012; Dittel & Vander Wall 2018). A number of studies have identified cache loss caused by sympatric competitors either at intra‐ or inter‐specific level (Vander Wall *et al*. 2006; Jansen *et al*. 2012; Gu *et al*. 2017). Unlike cache recovery by scatter‐hoarders that rely on both accurate spatial memory and olfaction (Yi *et al*. 2016a), pilferers, however, need highly sensitive olfaction to detect seeds buried by cache owners (Yi *et al*. 2016b). Therefore, seed trait‐mediated seed detectability of intra‐ and inter‐specific competitors is expected to play decisive but different roles in affecting seed hoarding preference of hoarding animals. Previous evidence shows that seeds with low nutritional value are less likely to be detected by sympatric animals (Barga & Vander Wall 2013). On the contrary, large seeds and seeds emitting strong odor are more likely to be detected by pilferers, causing great cache loss to scatter‐hoarding animals (Hollander *et al*. 2012; Yi *et al*. 2016a; Vander Wall *et al*. 2018). To avoid cache loss to cache pilferers, many scatter‐hoarding animals alter their hoarding preference in response to intra‐ and inter‐specific competition (Zhang *et al*. 2011; Yang *et al*. 2016; Cao *et al*. 2018). For example, large seeds with high nutritional value are cached farther, and fewer seeds are stored in single cache (Yi & Yang 2011), which is supposed to reduce seed detectability and cache loss.

Seed traits not only directly affect pre‐caching seed selection preference, but may also modify post‐caching seed detectability and therefore influence caching behavior of food hoarding animals. Although previous studies have investigated the effects of seed traits on hoarding behavior, the role of seed detectability in determining seed hoarding preference is largely neglected. Various aspects of seed traits (e.g. seed size, physical and chemical defense as well as nutrition status) may show profound effects on caching preference, while seed detectability appears to be mainly determined by seed odor rather than other seed traits (Longland & Dimitri 2018). Therefore, seed traits and seed detectability are expected to exhibit different or even contrasting effects on seed caching preference, although seed traits may also affect seed detectability of rodents. To date, however, study on the correlation of seed traits, seed detectability and seed hoarding preference of animals is still lacking. In the present study, the scatter‐hoarding animals Edward's long‐tailed rats (*Leopoldamys edwardsi*) and Chevrier's field mice (*Apodemus chevrieri*) were chosen to investigate whether the seed detectability of intra‐ and inter‐specific individuals influences seed hoarding preference of animals. We used seeds of 7 sympatric tree species (*Quercus variabilis*, *Choerospondias axillaris*, *Q. serrata*, *Q. acutissima*, *Cyclobalanopsis glauca*, *Lithocarpus harlandii*, and *Camellia oleifera*) showing contrasting seed traits to determine the correlation of seed traits, seed detectability and seed hoarding preference of the 2 rodent species. We expected that 1) seed traits will show consistent effects on seed hoarding preference of *L. edwardsi* and *A. chevrieri*; 2) seed detectability of *L. edwardsi* and *A. chevrieri* is dependent on seed traits; 3) seed detectability more than seed traits determines seed hoarding preference of *L. edwardsi* and *A. chevrieri*.

## METERIALS AND METHODS

### Study site

This study was conducted in the experimental enclosures in Dujiangyan city of Sichuan Province, China (31°4′N, 103°43′E) from September to December in 2018. The area belongs to a subtropical evergreen broad‐leaf forest (Chang & Zhang 2011). The local weather is common cloudy and foggy, average sunny hours is only 800–1000 h annually, and the relative humidity is generally more than 80%. The site is in a subtropical climate with an average annual temperature of 15.2°C, and an average annual rainfall of 1200–1800 mm (Yang 2018; Yang *et al*. 2019).

### Seed species used in this study

In autumn 2018, we collected fresh seeds of 7 tree species (*Q. variabilis*, *C. axillaris*, *Q. serrata*, *Q. acutissima*, *C. glauca*, *L. harlandii*, *C. oleifera*) directly from the ground after they ripen in the forest. Seeds of the uniform size of each species were selected for this experiment by using a water floating method to detect insect‐damaged and empty seeds. Previous studies have provided evidence that small rodents such as chestnut rats (*Niviventer fulvescens*), Edward's long‐tailed rats (*L. edwardsi*), white‐bellied rats (*Niviventer confucianus*), Chevrier's field mice (*A. chevrieri*) and south China field mice (*Apodemus draco*) consume and disperse seeds of the 7 tree species in the Dujiangyan area (Yang *et al*. 2019). Among these rodent species, *L. edwardsi* and *A. chevrieri* appear to be the predominant seed scatter‐hoarders.

### Rodent species used in this study

In the field, *L. edwardsi* and *A. chevrieri* are the dominant scatter‐hoarders that have a strong ability to pilfer caches. *N. confucianus* and *N. fulvescens* are generally larder‐hoarders, and are expected to exhibit poor pilferage abilities (Wang *et al*. 2018). Therefore, we selectively chose *L. edwardsi* and *A. chevrieri* as the focal animals in this study.

To capture the experimental animals, we used live traps (H × W × L: 30 cm × 15 cm × 15 cm) baited with peanuts or nuts and provisioned with dry leaves for keeping warm. The focal animals were brought back to the laboratory and caged individually. Prior to the behavioral experiments, all animals were fed individually in a plastic cage (H × W × L: 45 cm × 30 cm × 15 cm), and provide with rat chow (4% fat, 20% protein, 70% carbohydrate; Shenyang Maohua Biotechnology Co. Ltd., Liaoning, China) and tap water *ad libitum*. The temperature of feeding chamber was maintained at 20–25°C. After the behavioral experiments, all animals were released to the locations where they were originally captured.

### Semi‐natural enclosure

In this study, semi‐enclosures (10 m × 10 m × 1.5 m) were used to investigate the seed hoarding preference of the 2 rodent species by using seeds of 7 tree species in Dujiangyan area. The floor of the enclosures was paved with red bricks, but 64 shallow pits (24 cm × 12 cm × 6 cm) were evenly established. Fine sand was filled in each pit allowing rodents to cache in each enclosure. The top of enclosures was covered with transparent roof supported by a steel frame to prevent any predators from entering the enclosures. Two nesting boxes (40 cm × 40 cm × 40 cm) and 2 plates were placed at the corner of each enclosure to keep the experimental animals warm and provide adequate water for drinking. All animals were acclimatized in the enclosures for at least 2 nights before the behavioral experiments, during which they were provided peanuts and drinking water *at libitum*.

### Test of seed detectability

After acclimation, the animals were moved out of the enclosures and confined in the plastic cages temporarily. At 1800 hours, we cleaned each enclosure and buried 56 seeds (8 seeds of each species) randomly in the shallow pits (1 seed per pit). Seeds were buried 1–2 cm deep to mimic the average depth of caches established by rodents in the field (Xiao *et al*. 2008; Gu *et al*. 2017). Then, one focal animal was released into each enclosure immediately after establishment of artificial caches. The animals were allowed to detect and excavate the buried seeds for 14 h. At 0800 hours in the next morning, the focal animals were again moved out of the enclosures. All shallow pits were carefully checked to see how many artificially buried seeds were excavated for each seed species. In the seed detectability experiments, 10 adult *L. edwardsi* (6 ♂, 4 ♀, body weight: 448.7 ± 77.3 g, mean ± SD) and 10 adult *A. chevrieri* (7 ♂, 3 ♀, body weight: 33.64 ± 7.66 g, mean ± SD) were tested individually to see how seed traits affect seed detectability of hoarding rodents.

### Test of seed hoarding

Two days after acclimation, we placed 56 unlabeled seeds (8 seeds per tree species) at the center of each enclosure. Then, one focal animal was introduced into each enclosure at 1800 hours and allowed to move and forage freely for 14 h. At 0800 hours in the next morning, the subject animals were moved out of the enclosures and confined in the corresponding plastic cages. Then, the whole enclosure floor including all shallow pits were searched carefully to locate the intact seeds and seed debris. Seed fates were categorized into IIS (intact *in situ*), EIS (eaten *in situ*), IAR (intact after removal), EAR (eaten after removal), SH (scatter‐hoarded in the shallow pits) and LH (larder‐hoarded in the nesting boxes). In total, another 10 adult *L. edwardsi* (6 ♂, 4 ♀, body weight: 470.6 ± 70.9 g, mean ± SD) and 10 adult *A. chevrieri* (8 ♂, 2 ♀, body weight: 37.18 ± 3.81 g, mean ± SD) were tested individually in the seed hoarding experiments, to see how seed traits affect seed hoarding preference of rodents. Animals used in the seed detectability experiments were not re‐used in the seed hoarding experiments.

### Seed traits

Seed traits of the 7 tree species were cited from Yang (2018). Specifically, seed mass was measured by using an electronic scale to the nearest 0.01 g. Chemical analysis was conducted in duplicates on a mixture of healthy seeds for each tree species; Seed nutrient compositions were measured by Measure Center of Grain Quality, Ministry of Agriculture, China. Caloric values were calculated by the average gross‐energy equivalents of protein (17.2 KJ⋅g^−1^), fat (38.9 KJ⋅g^−1^) and carbohydrates (17.2 KJ⋅g^−1^), and caloric value per seed was then calculated by multiplying mean kernel dry mass by corresponding caloric value (Xiao *et al*. 2008).

### Statistical analyses

Graphed prism 9.0 was applied for data presentation. Preliminary analyses showed no significant relationship between seed chemical traits (e.g. protein, fat, starch, tannin and fiber) and hoarding and pilfering preference. Therefore, linear regression was used to assess the correlation between seed traits (seed mass, seed coat thickness and caloric value per seed), seed detectability and seed hoarding preference of the 2 rodent species. As seed traits include various aspects, for example seed size, thickness of seed coat, nutrients, and tannins, which may influence seed choice and hoarding by animals in a complicated way, we therefore combined seed physical and chemical aspects together into caloric value, in attempt to obtain convincible relationships between seed traits and seed detectability and hoarding preference of the 2 rodent species. Caloric values per seed were log transformed before linear regression.

## RESULTS

### Correlation of seed traits to seed hoarding preference of *L. edwardsi* and *A. chevrieri*

**Figure 1:**
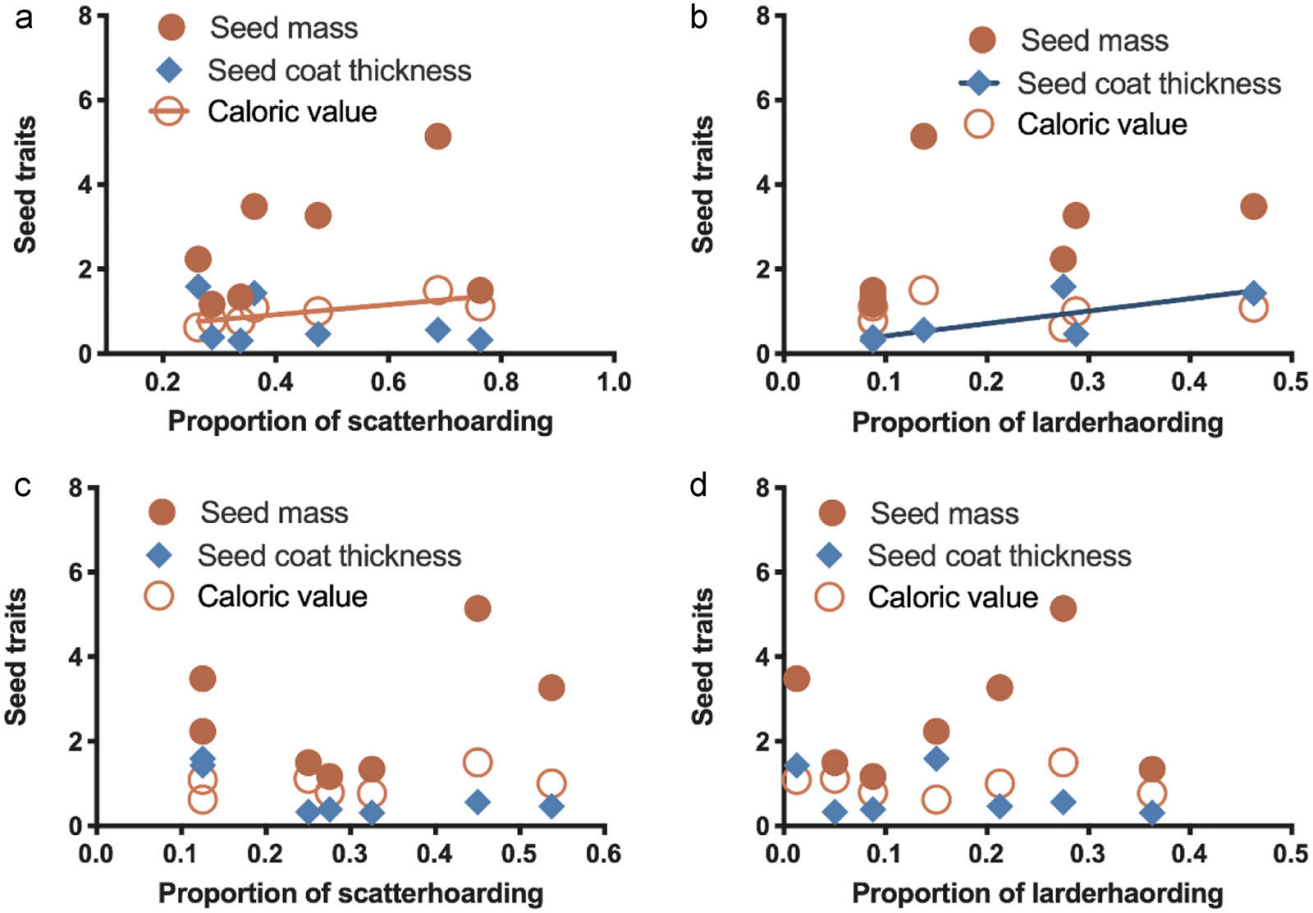
Correlation of seed traits with scatter‐hoarding and larder‐hoarding preference of *Leopoldamys edwardsi* (a and b) and *Apodemus chevrieri* (c and d).

Seed selection and hoarding preference by *L. edwardsi* and *A. chevrieri* were summarized in Table 1. Linear regression indicated that no significant correlation was detected between seed mass and seed coat thickness with scatter‐hoarding preference of *L. edwardsi* (seed mass: *r* ^2^ = 0.4484, *P* = 0.0999; seed coat thickness: *r* ^2^ = 0.073, *P* = 0.5576; Fig. 1a). Seed mass and caloric value showed no significant effect on seed larder‐hoarding preference of *L. edwardsi* (seed mass: *r* ^2^ = 0.180, *P* = 0.3430; caloric value: *r* ^2^ = 0.0001, *P* = 0.9939; Fig. 1b). But caloric value and seed coat thickness showed effect on scatter‐ and larder‐hoarding preference respectively (caloric value: *r* ^2^ = 0.610, *P* = 0.038; *r* ^2^ = 0.606, *P =* 0.0393; Fig. 1a, b).

**Table 1:** Seed choice and seed preference (%) by *Leopoldamys edwardsi* (*Le*) and *Apodemus chevrieri* (*AC*)

		IIS	EIS	IAR	EAR	LH	SH	PR
*Le*	*Qs*	20	33.75	0	8.75	8.75	28.75	34.72
	*Qv*	0	8.75	0	7.5	13.75	68.75	11.11
	*Qa*	1.25	12.5	0	10	28.75	47.5	26.38
	*Cg*	7.5	28.75	0	18.75	8.75	33.75	29.17
	*Ca*	0	0	0	11.25	46.25	36.25	33.33
	*Co*	0	0	1.25	11.25	8.75	76.25	23.61
	*Lh*	35	1.25	1.25	3.75	27.5	26.25	29.17
*AC*	*Qs*	55	2.5	0	6.25	8.75	27.5	43.06
	*Qv*	23.75	0	2.5	0	27.5	45	59.72
	*Qa*	25	0	0	0	21.25	53.75	47.22
	*Cg*	16.25	0	2.5	12.5	36.25	32.5	77.77
	*Ca*	82.5	0	3.75	0	1.25	12.5	23.61
	*Co*	70	0	0	0	5	25	33.33
	*Lh*	68.75	0	3.75	0	15	12.5	52.78

*Qs*, *QV*, *Qa*, *Cg*, *Ca*, *Co* and *Lh* stand for *Quercus serrata*, *Q. variabilis*, *Q. acutissima*, *C. glauca*, *Choerospondias axillaris*, *Lithocarpus harlandii* and *C. oleifera*, respectively. IIS, EIS, IAR, EAR, LH, SH, and PR stand for seeds intact *in situ*, eaten *in situ*, intact after removal, eaten after removal, larder‐hoarded, scatter‐hoarded, and pilferage rate, respectively. Data are shown as percentage.

Similarly, seed traits, for example seed mass, caloric value and seed coat thickness, did not showed effects either on scatter‐hoarding preference of *A. chevrieri* (seed mass: *r* ^2^ = 0.142, *P* = 0.4046; seed coat thickness: *r* ^2^ = 0.463, *P* = 0.0925; caloric value: *r* ^2^ = 0.192, *P* = 0.3248; Fig. 1c), or on larder‐hoarding preference of *A. chevrieri* (seed mass: *r* ^2^ = 0.024, *P* = 0.7403; seed coat thickness: *r* ^2^ = 0.153, *P* = 0.3859; caloric value: *r* ^2^ = 0.0005, *P* = 0.9607; Fig. 1d).

Neither seed protein, fat, starch, fiber nor tannin of the 7 tree species alone could explain seed hoarding preference of *L. edwardsi* and *A. chevrieri* (data not shown here but all *P* > 0.05).

### Correlation of seed detectability to seed hoarding preference of *L. edwardsi* and *A. chevrieri*

**Figure 2:**
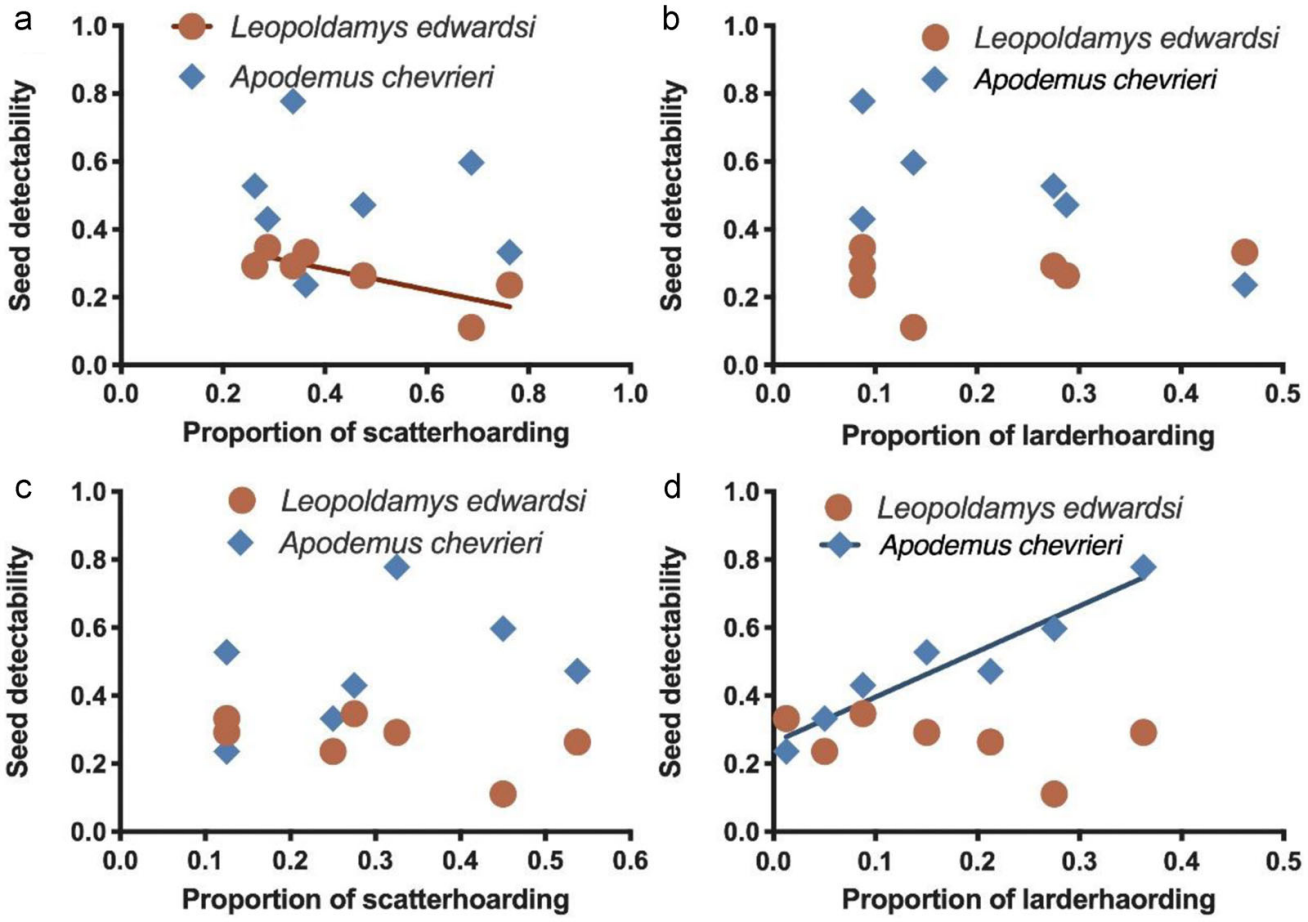
Correlation of scatter‐hoarding and larder‐hoarding preference of *Leopoldamys edwardsi* (a and b) and *Apodemus chevrieri* (c and d) with seed detectability of *Leopoldamys edwardsi* and *Apodemus chevrieri*.

Our results showed a significant positive correlation between seed detectability of *L. edwardsi* and their own scatter‐hoarding preference (*r* ^2^ = 0.604, *P =* 0.0397; Fig. 2a). However, no significant correlation was detected between seed detectability of *A*. *chevrieri* and scatter‐hoarding preference of *L. edwardsi* (*r* ^2^ = 0.022, *P =* 0.7507; Fig. 2a). Seed detectability of either *L. edwardsi* or *A. chevrieri* showed no significant effect on larder‐hoarding preference of *L. edwardsi* (*r* ^2^ = 0.090, *P* = 0.5137; *r* ^2^ = 0.274, *P* = 0.2280; Fig. 2b). Scatter‐hoarding preference of *A. chevrieri* was not correlated with seed detectability of either *A. chevrieri* (*r* ^2^ = 0.296, *P* = 0.2065) or *L. edwardsi* (*r* ^2^ = 0.161, *P* = 0.3717, Fig. 2c). There was significant correlation between larder‐hoarding preference of *A. chevrieri* and its own seed detectability (*r* ^2^ = 0.914, *P* = 0.0008, Fig. 2d). Seed detectability of *L. edwardsi*, however, did not influence larder‐hoarding preference of *A. chevrieri* (*r* ^2^ = 0.182, *P* = 0.3392, Fig. 2d).

**Figure 3:**
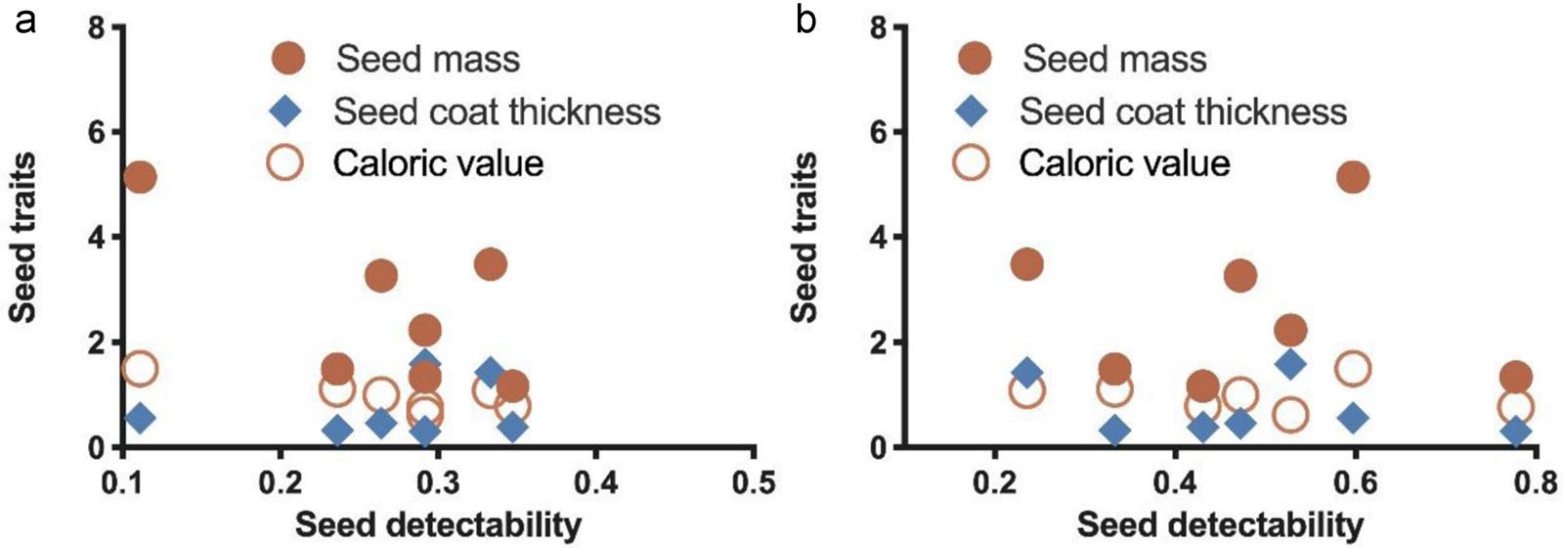
Correlation of seed traits with seed detectability of *Leopoldamys edwardsi* (a) and *Apodemus chevrieri* (b).

In addition, seed detectability either of *L. edwardsi* or *A. chevrieri* was not correlated with seed mass, seed coat thickness and caloric values (all *P* > 0.05; Fig. 3a, b). We also failed to detect any correlation between seed detectability of *L. edwardsi* and *A. chevrieri* with the specific seed nutrients, for example crude protein (*r* ^2^ = 0.008, *P* = 0.8425; *r* ^2^ = 0.0001, *P* = 0.9862), crude fat (*r* ^2^ = 0.004, *P* = 0.8970; *r* ^2^ = 0.0410, *P* = 0.6634), crude starch (*r* ^2^ = 0.0002, *P* = 0.9787; *r* ^2^ = 0.0371, *P* = 0.6790), crude fiber (*r* ^2^ = 0.0137, *P* = 0.8025; *r* ^2^ = 0.006, *P* = 0.8662) and tannins (*r* ^2^ = 0.1269, *P* = 0.4330; *r* ^2^ = 0.4639, *P* = 0.0920), respectively.

## DISCUSSION

Seed traits are usually considered as the main differences between tree species (Lichti *et al*. 2017). Many seed traits have been identified to affect the hoarding preference of rodents, including seed size (Wang & Chen 2009; Jansen *et al*. 2012; Wróbel & Zwolak 2017), thickness of seed coat (Zhang & Zhang 2008; Chang *et al*. 2012), nutritional value (Xiao *et al*. 2006) and secondary compounds (Xiao *et al*. 2008; Wang & Chen 2009; Perea *et al*. 2011). In our study using multiple seed species, however, seed trait appears to be an unimportant role in determining either of scatter‐ or larder‐hoarding preference of *L. edwardsi* and *A. chevrieri*, despite the effect of caloric value and seed coat thickness on hoarding preference of *L. edwardsi*. Previous studies have provided evidence that seed traits, such as seed size, seed chemistry and seed coat thickness play a decisive role in affecting seed hoarding preference of various rodents (Vander Wall 2010; Galetti *et al*. 2015; Sundaram *et al*. 2018), and even mediate the evolution of hoarding behaviors of animals (Wang & Chen 2009; Cao *et al*. 2018). This inconsistency with our results may be attributed to the fact that previous studies often focus on a single plant species to explore the impacts of seed traits on hoarding preference of rodents at population level (Cao *et al*. 2018). While in our study, the effect of seed traits was tested on seed hoarding preference of 2 rodent species by using multiple plant species that coexist and show similar fruit ripening periods (Yang 2018; Yang *et al*. 2019). It is well accepted that different seed traits of a given species co‐vary to influence seed preference of granivorous rodents; therefore teasing apart the actual effect of single seed trait appears to be difficult (Gong *et al*. 2015; Zhang *et al*. 2016). Our study using multiple tree species, accounting for the integrative effect of seed caloric value, has the potential to reduce the interactive effects of seed traits of hoarding preference. Our results may convey the information that seed hoarding preference of rodents is based on an integration of various seed traits rather than a single seed property.

Seeds cached by food‐hoarding animals can either be recovered by the hoarders or pilfered by naïve competitors (Zhang *et al*. 2016; Dittel & Vander Wall 2018). Cache owners have been recognized to have a cache recovery advantage over pilferers due to the accurate spatial memory on their own caches, which is absent in cache pilferers that rely only on olfaction to excavate seeds from caches (Yi *et al*. 2016b; Gu *et al*. 2017). Relying on this advantage, cache owners can acquire a great number of stored seeds, although many caches will be pilfered by other competitors (Gu *et al*. 2017). In this study, we established artificial caches and allowed rodents to detect the buried seeds by using olfaction. Contrary to our expectation, we failed to find any correlation between seed traits with seed detectability either of *L. edwardsi* or *A. chevrieri*, indicating that burial may conceal seed physical traits and nutritional value and then hinder seed selection preferences of the pilferers when excavating the cached seeds (but see Vander Wall *et al*. 2018). Evidence shows that the release of volatile compounds from seeds determines seed detectability of rodents (Longland & Dimitri 2018). Although we failed to measure the differences in seed odor emission of the 7 tree species, seed volatiles may interact with other seed traits (e.g. seed mass or nutritional value) and manipulate seed detectability of rodents in our study. Despite this, our study presented evidence that seed detectability of *A. chevrieri* was only positively correlated with their larder‐hoarding preference. Moreover, seed scatter‐hoarding preference of *L. edwardsi* was affected only by intra‐specific seed detectability, reflecting that seed hoarding preference is mainly affected by intra‐specific rather than inter‐specific seed detectability. Seed detectability of inter‐specific pilferers did not show any influence on seed hoarding preferences of *L. edwardsi* and *A. chevrieri*, implying that cache loss to inter‐specific competitors is probably not an important selective pressure on seed hoarding strategies. Our results may provide some evidence that cache loss to intra‐specific pilferers contributes to evolution of seed hoarding preference although only 2 rodent species were investigated. In our study, however, seed scatter‐hoarding preference of *L. edwardsi* and larder‐hoarding preference of *A. chevrieri* were influenced by intra‐specific seed detectability. This difference can be explained by the facts that *L. edwardsi* are predominant scatter‐hoarders while *A. chevrieri* are predominant larder‐hoarders although they both scatter‐ and larder‐hoard seeds in the study area (Zhang *et al*. 2008; Chang & Zhang 2011; Yi *et al*. 2021). Our results showing that seeds scatter‐hoarded were 3.86 times as those larder‐hoarded by *L. edwardsi*, whereas seeds larder‐hoarded were 1.12 times as those scatter‐hoarded by *A. chevrieri* further support this speculation (Table 1). Another possible explanation is that scatter‐hoarding intensity is positively related to seed detectability of rodents (Vander Wall *et al*. 2009; Wang *et al*. 2018), and therefore *L. edwardsi* mainly modify their scatter‐hoarding preference to a given seed species while *A. chevrieri* exhibiting lower seed detectability tend to manage seeds in their larders in response to the risk of cache loss.

Although it has been documented that hoarding animals, such as seed‐caching birds and rodents, modify their caching behaviors in the presence of intra‐ or inter‐specific cache thieves (Steele *et al*. 2008; Galvez *et al*. 2009; Hirsch *et al*. 2012), our study may provide convincible evidence that intra‐ rather than inter‐specific seed detectability exerts impacts on seed hoarding preference of food‐hoarding animals. It is possible that the seed caching rodents in our study assessed the risk of seed pilferage by intra‐specific thieves and then modified their hoarding preference towards a given seed species. *Leopoldamys edwardsi* may scatter‐hoard more seeds that are less likely to be excavated from caches by its conspecifics, while *A. chevrieri* tend to larder‐hoard more seeds that are more likely to be detected from caches by the individuals of its kind. The 2 rodent species respond to the perceived cache loss risks and manage the stored seeds according to intra‐specific seed detectability, representing an evolutionary adaptation of food‐hoarding animals to plants producing propagules with contrast seed traits, which would allow them to gain more rewards from their stored seeds.

## CONFLICT OF INTEREST

The authors declare no conflict of interest.

## AUTHOR CONTRIBUTIONS

XY conceived and designed the experiments. MW performed the experiments. MW and XY analyzed the data. MW and XY wrote the manuscript.
